# The Relationship Between Serum Levels of Thyroglobulin Antibody and the Risk of Recurrence in Patients With Differentiated Thyroid Cancer

**DOI:** 10.1002/cnr2.70191

**Published:** 2025-05-27

**Authors:** Amirian Fatemeh, Yaghoubi Mohammad Ali, Mehrad‐Majd Hassan, Mohebbi Masoud, Layegh Parvin, Vazifeh‐Mostaan Leila, Dadgar Moghaddam Maliheh, Amirian Zahra, Taghavi Ghazaleh

**Affiliations:** ^1^ Faculty of Medicine, Department of Internal Medicine Mashhad University of Medical Sciences Mashhad Iran; ^2^ Metabolic Syndrome Research Center Mashhad University of Medical Sciences Mashhad Iran; ^3^ Cancer Molecular Pathology Research Center Mashhad University of Medical Sciences Mashhad Iran; ^4^ Clinical Research Development Unit Ghaem Hospital, Faculty of Medicine, Mashhad University of Medical Sciences Mashhad Iran; ^5^ Endocrine Research Center Mashhad University of Medical Sciences Mashhad Iran; ^6^ Endocrinology & Metabolism Member of Metabolic Syndrome Research Center (MSRC) Mashhad University of Medical Sciences Mashhad Iran; ^7^ Mashhad University of Medical Sciences Mashhad Iran; ^8^ Community Medicine, Community Medicine Department School of Medicine Mashhad Iran; ^9^ University of Medicine, Trauma and General Surgery Wards, Imam Ali Hospital Zahedan Iran

**Keywords:** differented thyroid cancer, Tg Ab, thyroid cancer

## Abstract

**Objective:**

Thyroid Autoantibodies (TgAbs) are associated with autoimmune thyroid disorders and are also used in thyroid cancer follow‐up to monitor for recurrence of disease. This study aimed to explore the potential utility of TgAbs as a surrogate tumor marker and examine the relationship between fluctuations in TgAbs levels and disease recurrence in patients with Differentiated Thyroid Cancer (DTC).

**Methods:**

This cohort study was conducted on a sample of 97 patients who underwent thyroidectomy for differentiated thyroid cancer (DTC) between the years 2017 and 2021. Following surgery (with or without lymph node dissection), levothyroxine therapy and 131 iodine were prescribed (as necessary). Regular laboratory evaluations were conducted, which involved measuring Tg and TgAb at 3 and 6 months postoperatively. Patients were classified based on recurrence rate and different levels of TgAb, and ROC analysis was applied. All data were analyzed with SPSS24, and a *p*‐value < 0.05 was considered statistically significant.

**Results:**

For low‐risk patients, TgAb trends over time showed an increase at 6 months, while for high‐risk patients, TgAb levels continuously rose starting from 3 months. Although TgAb levels above the functional sensitivity threshold did not predict recurrence overall, changes in TgAb levels at 6 months compared to 3 months after surgery were indicative of recurrence.

**Conclusion:**

In the entire population, having TgAb levels higher than the functional sensitivity threshold had no risk of various relapses. However, changes in TgAb serum levels at 6 months after surgery, compared to 3 months after surgery, can serve as an indication of tumor recurrence.

## 1 Introduction

Papillary Thyroid Carcinoma (PTC) and Follicular Thyroid Carcinoma (FTC) represent the predominant forms of well‐differentiated thyroid cancers (DTC), encompassing the majority of thyroid cancer diagnoses. PTC is more prevalent among women in their forties and fifties. Notably, though the overall incidence of thyroid cancer has been increasing, the mortality rate has remained relatively constant, hovering around 0.5 per 100 000 individuals from 2003 to 2012 [1, 2].

To effectively monitor patients with DTC following thyroidectomy, Thyroglobulin (Tg) serves as a valuable biomarker for evaluating treatment response and early detection of recurrence; however, Thyroglobulin antibody (TgAb) plays a more crucial role in post‐treatment monitoring because of its potential interference with thyroglobulin levels [3, 4]. Changes in TgAb levels can be used as an indicator for tumor recurrence during follow‐up evaluations, even in patients who initially test negative for TgAb but later test positive during subsequent assessments [5, 6].

To accurately measure TgAb, it is recommended to use immunoassay methods that consider both the cut‐off value determined by the manufacturing company (MCO) and functional sensitivity (FS). Reference values for TgAb have been established to differentiate individuals with autoimmune thyroid disease from those without it; however, detectable concentrations above FS or even below MCO's cut‐off point (referred to as borderline) can interfere with serum Tg levels. The variation of TgAb can provide valuable insights into the presence of persistent or recurrent disease after treatment.

Immunometric assays (IMA) are currently the most widely used tests for measuring Tg levels. However, patients with serum Tg‐IMA concentrations face a significant challenge in accurately determining whether these findings indicate complete remission or if interference from TgAb leads to an underestimation of actual levels [7, 8].

The impact of anti‐Tg antibodies on the decision to administer I‐131 after thyroidectomy is still inadequately studied. While I‐131 treatment offers the advantage of removing thyroid tissue, which serves as an antigen source for TgAb, its prospective evaluation and impact on clinical outcomes require further investigation. Present data do not support the hypothesis that administering I‐131 solely based on the presence of TgAb is a viable approach; instead, it should be considered alongside other clinical and pathological factors [9].

In cases where alternative methods are lacking, neck ultrasound and other imaging findings form the foundation for assessing disease risk classification. An increase in TgAb level confirms cancer progression and necessitates further evaluation, while a decrease may correspond to reduced tumor burden. Stable levels warrant imaging assessments depending on individual clinical conditions [10]. Some researchers have proposed using TgAb as a surrogated tumor marker during long‐term follow‐up in DTC patients as an indication of disease recurrence. Nonetheless, this remains a controversial topic due to potential underdetection of TgAb and an absence of clear guidelines regarding changes in its levels over time. The issue surrounding interference between TgAb and thyroglobulin continues to be classified as “indeterminate response to treatment” when clinically evaluating patients with thyroid cancer [11].

The aim of this study is to evaluate the role of TgAb and its potential as a surrogate tumor marker by examining correlations between changes in TgAb levels and disease recurrence in patients with DTC.

## 2 Methods

In this retrospective cohort study, all patients who underwent surgery for DTC (follicular and papillary) from 2017 to 2022 were included. This study was approved by the Mashhad University of Medical Sciences Organizational Ethics Committee (IR.MUMS.MEDICAL.REC 1401.095) and informed consent was obtained from all patients.

The inclusion criteria for this study consisted of:

1. Confirmed diagnosis of DTC and a history of thyroidectomy with or without neck dissection.
2. Receiving 131I (RAI) treatment.
3. A minimum of 12 months of available patient data.

Exclusion criteria were:

1. Unwillingness to participate in the study.
2. A lack of specific pathology.
3. Patients diagnosed with Hashimoto's thyroiditis.
4. Patients with extremely high levels of TgAb (Avoiding scattered data).
5. Inadequate follow‐up data.

Central or lateral lymph node dissection, levothyroxine, and adjuvant iodine‐131 therapy were also prescribed based on the indications. Patient characteristics (gender and age), ultrasound findings, tumor characteristics (papillary or follicular), and treatment type (total thyroidectomy versus lobectomy/received versus not received iodine therapy) were documented. Data related to the course of the disease during the follow‐up period was documented on a researcher‐made checklist. The laboratory evaluations included the measurement of Tg and TgAb levels at least every 3 to 6 months.

Disease recurrence was based on the following criteria:

1. Structural findings in ultrasound.
2. Image findings in whole‐body scan (if performed).
3. Malignant cells in fine‐needle aspiration biopsy (FNA).
4. Tissue examination during the second operation.

The risk of disease recurrence was classified and recorded based on the criteria set by the American Thyroid Association (ATA) in the 2015 guidelines and defined as low risk, intermediate risk, and high risk [12]. Disease recurrence was confirmed by cytology, histology, or post‐therapy 131I diagnostic scan.

Among these patients who had 1 year of follow‐up and were measured at least two tests of Tg and TgAb (with or without levothyroxine), at intervals of at least 3–6 months after surgery, were included in this study. Patients with TgAb levels below functional sensitivity (FS) during follow‐up were excluded from the study.

FS is defined as the lowest concentration of TgAb that can be measured with a coefficient of variation < 20%, 10 UI/mL [4]. Serum levels equal to or greater than 10 IU/mL (higher than FS) in the measured samples were considered positive. Considering that TgAb may increase transiently after surgery or treatment with I131, it was measured at least 3 months after treatments. Based on changes in TgAb serum concentration, one of the following five different patterns was defined for patients:

1. Stable positive TgAb concentration with less than 50% changes during follow‐up (every 6 months for 2 years).
2. Increase in TgAb level above FS (from negative to positive).
3. The trend of continuous increase of TgAb (increase of more than 50%).
4. TgAb level decreases below FS or TgAb decreases from positive to negative.
5. TgAb reduction level of more than 50%.

Then, the response to the treatment was evaluated both biochemically and structurally using the Tg serum level with or without levothyroxine. Responses were categorized as excellent response, indeterminate response, and incomplete response. The “response to treatment” ranges in patients treated with levothyroxine were Tg serum level less than 0.2, 0.2–1, and more than 1, respectively. In patients without levothyroxine therapy, the ranges were less than 1, 1–10, and more than 10, respectively.

All high‐risk patients have received radioiodine. In studies, it has been stated that not all intermediate patients are necessarily required to take radioiodine, and it is prescribed based on various indications, and intermediate risk is not the only criterion for prescribing radioiodine [13, 14].

## 3 Statistics

Data was collected and entered into SPSS version 24 software, by means of statistical methods, and the relationship between TgAb and disease recurrence or stability was compared. Because of the non‐normal distribution, nonparametric tests including Repeated measures, Mann–Whitney U, and chi‐square were utilized for analyses. Descriptive statistics were reported at a significance level of α = 0.05. The receiver operating characteristic curve has been used to evaluate the clinical value of baseline (before or right after surgery) serum TgAb and Tg in predicting the risk of recurrence by determining the sensitivity and specificity as well as determining the cut‐off point.

## 4 Results

A total of 97 patients (19) males (19.6%) and 78 females (80.4%) with a history of DTC participated in the study. The mean age was 37.77 ± 9.43 years. Only one patient had a history of radiation exposure, and 6 patients (6.2%) had a family history of thyroid cancer. The mean serum vitamin D level was 30.69 ± 13.48 mg/dL. The number of patients with recurrence was 14 (14.4%) and the number of patients with no recurrence was 83 (85.6%). Demographic findings and follow‐up data for the patients are presented in Table 1.

**TABLE 1:** Patient demographic information.

Variable		*N*	%
Gender	Male	19	19.6
	Female	78	80.4
History of neck radiation	Yes	1	1
	No	96	99
Familial history of thyroid cancer	Yes	6	6.2
	No	91	93.8
type DTC	Papillary	89	91.8
	Follicular	8	8.2
Total thyroidectomy	Yes	87	89.7
	No	10	10.3
Lymph node dissection	No	44	45.4
	Central	26	26.8
	Lateral	7	7.2
	Central & Lateral	20	20.6
Post‐operative Levothyroxin treatment	Yes	97	100
	No	0	0
treatment I‐131	Yes	22	22.7
	No	75	77.3
Risk stratification	Low Risk	64	66
	Intermediate Risk	23	23.7
	High Risk	10	10.3
Recurrence rate	Yes	14	14.4
	No	83	85.6

The ultrasound findings revealed that 64 patients (66%) had hypoechoic nodules, 51 patients (52.6%) had microcalcifications, 9 patients (9.3%) showed central vascularity, 17 patients (17.5%) exhibited peripheral asymmetry, and 40 patients (41.2%) displayed lymphadenopathy. Thyroid nodules with microcalcifications, central vascularity, and lymphadenopathy are indicative of potential malignancy (Table 2).

**TABLE 2:** Patient ultrasound findings.

Sonographic findings	Hypo‐echogenicity	Microcalcification	Central vascularity	Peripheral asymmetry	Lymphadenopathy
%	6565%)(	51 (51%)	9 (9%)	17 (17%)	40 (40%)

**FIGURE 1:**
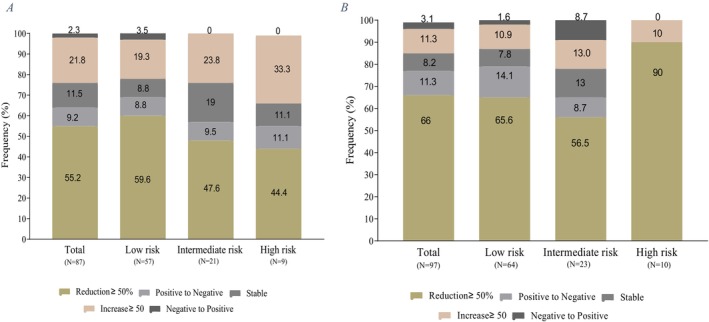
TgAb changes after the surgery. A. TgAb changes after the 3 months of surgery. B. TgAb changes after the 6 months of surgery.

Compared to 3 months post‐surgery, changes in TgAb levels at 6 months were significantly associated with tumor recurrence (*p* = 0.01 and *p* = 0.02, respectively). Specifically, patients with > 50% decline in TgAb at 3 months followed by > 50% increase at 6 months had higher recurrence rates (*p* = 0.01). Therefore, TgAb trends at 6 months post‐surgery may be most indicative of recurrence risk (Figure 1).

In order to evaluate the clinical value of serum TgAb levels and Tg levels in predicting the risk of recurrence and evaluating the potential of their diagnostic use by determining the sensitivity and specificity as well as determining the cut‐off point, the ROC curve has been used. The results related to the area under the curve, the best cut point, and the sensitivity and specificity of the two studied markers are provided (Tables 3 and 4).

**TABLE 3:** The results of ROC analysis to estimate the best cut‐off, sensitivity, and specificity of TgAb levels at baseline, 3 months post‐surgery, and 6 months post‐surgery in predicting the risk of recurrence.

Biomarker	*p*	Area under the curve ROC (95% CI)	Best cut‐off	Sensitivity %	Specificity %
TgAb (Baseline)	0.298	0.592 (0.40–0.79)	23	69	46
TgAb (3 Months)	0.175	0.618 (0.44–0.80)	11.3	62	40
TgAb (6 months)	0.004	0.748 (0.61–0.89)	17.5	77	67

**TABLE 4:** The results of ROC analysis to estimate the best cut‐off, sensitivity, and specificity of Tg levels at baseline, 3 months post‐surgery, and 6 months post‐surgery in predicting the risk of recurrence.

Biomarker	*p*	Area under the curve ROC (95% CI)	Best cut‐off	Sensitivity %	Specificity %
Tg (Baseline)	< 0.001	0.850 (0.74–0.96)	3.5	80	85
Tg (3 months)	0.022	0.692 (0.52–0.86)	0.055	71	60
Tg (6 months)	0.001	0.779 (0.64–0.92)	0.20	50	94

**FIGURE 2:**
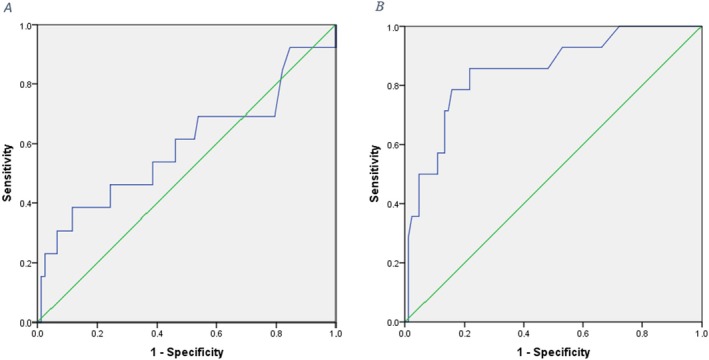
A. ROC curve to evaluate the sensitivity and specificity of baseline TgAb. B. ROC curve to evaluate the sensitivity and specificity of baseline Tg.

The results of ROC analysis showed that the best cutoff point for TgAb to differentiate between people with and without relapse is equal to 23 mg/dL. The sensitivity and specificity of TgAb in differentiating people for high and low risk for recurrence were reported as 69% and 46%, respectively. These values indicate the lack of clinical significance of the TgAb serum level before or right after the surgery (Figure 2A).

The ROC analysis for Tg showed a cutoff point equal to 3.5 mg/dL for differentiating people with high and low risk of recurrence. The sensitivity and specificity of Tg in differentiating people with high and low risk for relapse were reported as 80% and 85%, respectively. Considering the level under the curve equal to 0.85, the serum Tg level has a great clinical value in predicting the risk of recurrence of patients (Figure 2.B).

**FIGURE 3:**
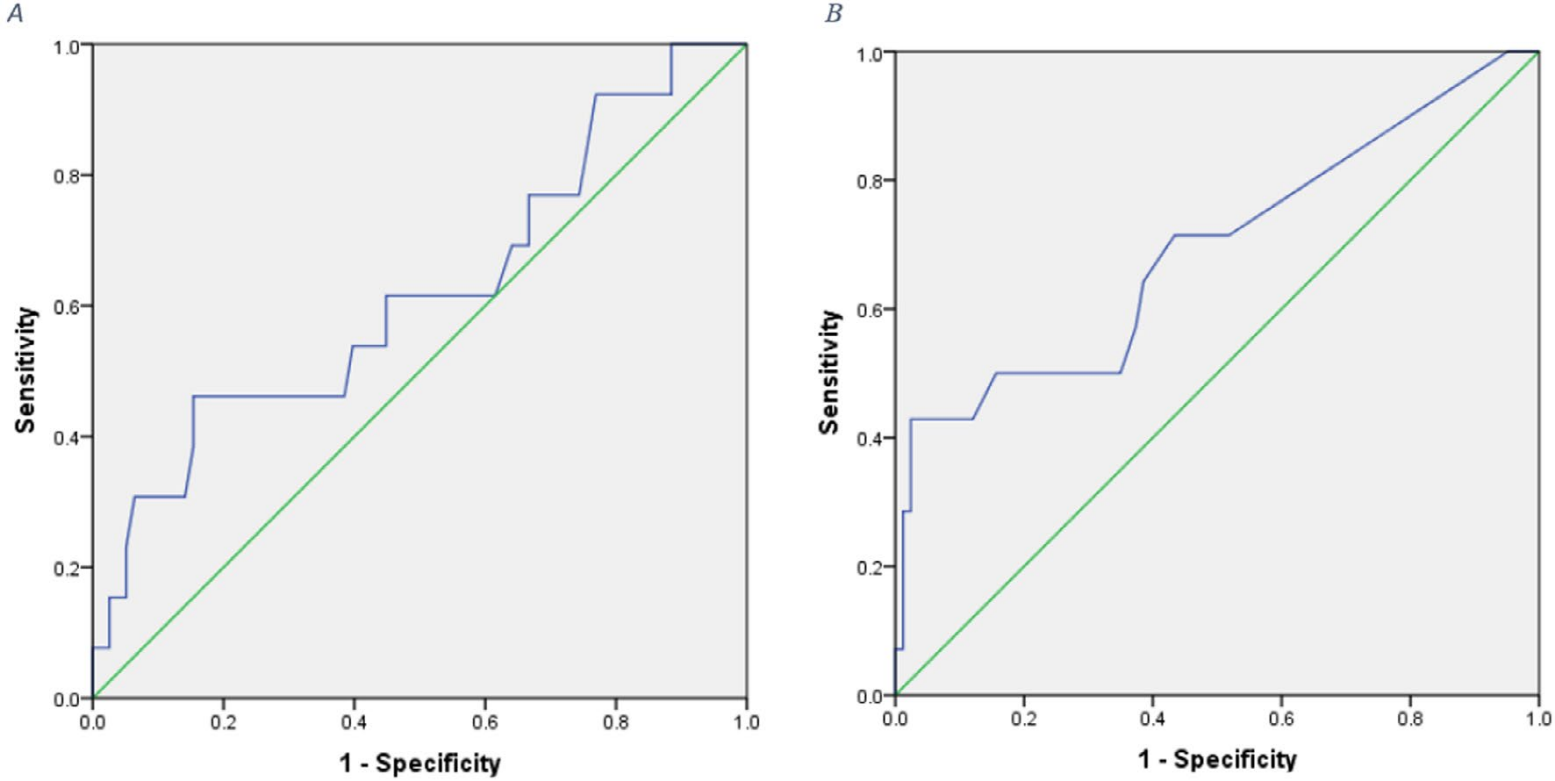
A. ROC curve to evaluate the sensitivity and specificity of TgAb measured 3 months after surgery. B. ROC curve to evaluate the sensitivity and specificity of Tg measured 3 months after surgery.

At three months post‐surgery, the best cut‐off point for TgAb to differentiate between people with and without relapse is equal to 11.3 mg/dL. The sensitivity and specificity of TgAb in differentiating people for high and low risk for recurrence were reported as 62% and 40%, respectively. Considering the level under the curve equal to 0.61 and the non‐significance of the *p* value, TgAb serum level measured 3 months after surgery has no clinical significance to predict the recurrence(Figure 3.A).

The ROC analysis for Tg showed a cutoff point equal to 0.055 mg/dL for differentiating people with high and low risk of recurrence. The sensitivity and specificity of Tg in differentiating people with high and low risk for relapse were reported as 71% and 60%, respectively. Considering the area under the curve equal to 0.69, the serum Tg level has an acceptable clinical value in predicting the risk of relapse (Figure 3B).

**FIGURE 4:**
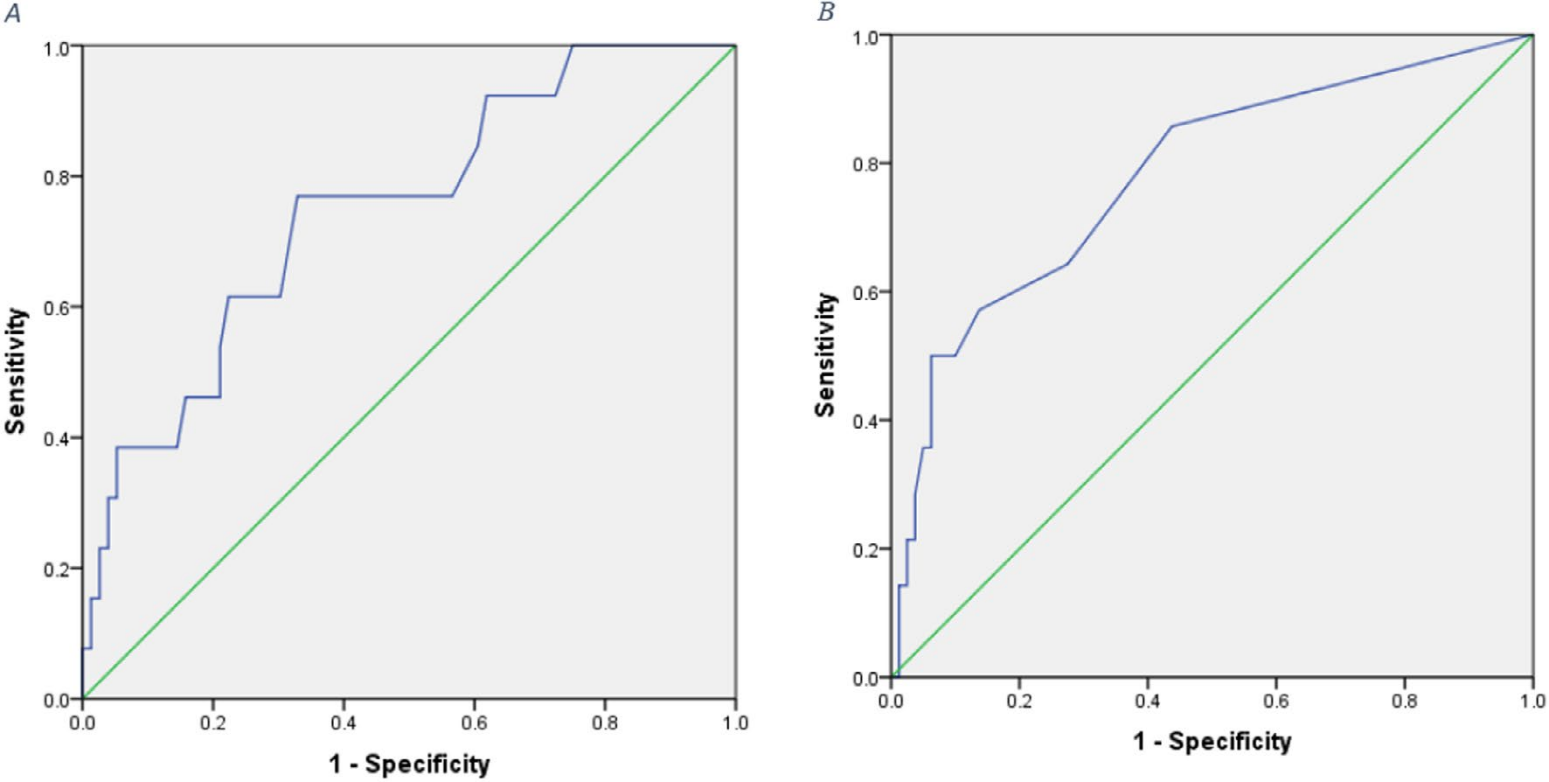
A. ROC curve to assess the sensitivity and specificity of TgAb measured 6 months after surgery. B. ROC curve to assess the sensitivity and specificity of Tg measured 6 months after surgery.

The results of ROC analysis showed that the best cut‐off point for TgAb levels six months post‐surgery to differentiate between people with and without relapse is equal to 17.5 mg/dL. The sensitivity and specificity of TgAb in differentiating people for high and low risk for recurrence were reported as 77% and 67%, respectively. Considering the level under the curve equal to 0.75, the serum level of TgAb measured 6 months after surgery has an acceptable clinical value in predicting the risk of recurrence of patients (Figure 4A).

The ROC analysis for Tg showed a cutoff point equal to 0.20 mg/dL for differentiating people with high and low risk of recurrence. The sensitivity and specificity of Tg in differentiating people with high and low risk for relapse were reported as 50% and 94%, respectively. Considering the level under the curve equal to 0.78, the serum Tg level has an acceptable clinical value in predicting the risk of relapse of patients (Figure 4.B).

We compared changes in serum levels of Tg and TgAb (baseline and 6 months postoperative) using ROC analysis, between patients who received radioactive iodine and those who did not receive radioiodine, as well as patients who underwent lobectomy with those who underwent total thyroidectomy. The results did not change in the incidence of recurrence and response to treatment, which means that lobectomy or radioiodine intake did not interfere with TgAb and Tg levels.

## 5 Discussion

In this study, the baseline serum level of Tg is a significantly clinical indicator of tumor recurrence. However, the TgAb baseline serum level has no such clinical significance. At 3 months after surgery, Tg levels were statistically significant in the risk of recurrence, while the course of TgAb serum level was not a predictor of recurrence at 3 months after surgery. Tg and TgAb serum levels 6 months after surgery can indicate tumor recurrence. From a practical point of view, our findings are not applicable for predicting recurrence beyond the first year after thyroidectomy in patients without recurrence at the end of the first year.

Comparison with similar studies revealed that TgAb is present in approximately 25% of patients with DTC during their initial evaluation following surgery. Even relatively low detectable concentrations of TgAb interfere with the measurement of Tg [4].

In a study by Kim et al. that evaluated 824 DTC patients who underwent total thyroidectomy and 131I ablation with undetectable Tg levels between 6 and 12 months post‐surgery and 131I treatment from 1995 to 2003 in Korea, the researchers found, consistent with the present study, that serum TgAb levels assessed 6 to 12 months after surgery and I131 ablation can predict recurrence in patients with undetectable Tg. Furthermore, in patients with undetectable Tg and positive TgAb levels or a reduction of less than 50%, changes in TgAb concentrations at 6–12 months after surgery may serve as a prognostic indicator of recurrence [15].

Latrofa et al. (2016) compared serum Tg and TgAb levels in 177 DTC patients who underwent total thyroidectomy and 131I ablation. In line with our study, they demonstrated that low TgAb levels as determined by the IMA method in those with undetectable Tg can rule out metastatic disease [16].

Barashki et al. (2023) conducted a retrospective study on 710 DTC patients in terms of time to complete response, recurrence rate, one‐year response, and potential correlation between pre‐surgery TSH levels and initial disease stage. Consistent with the present study, most patients had papillary cancer, and the mean patient age was similar [17].

Latour et al. (2023) compared initial postoperative Tg with recurrence in 143 DTC patients who underwent total thyroidectomy and radioactive iodine from 2011 to 2021. Though the population mean age was approximately 5 years older than our study, consistent with our study, papillary histology predominated. Tg immediately following surgery had a significant association with patient recurrence risk, which was consistent with our investigation [18].

Kaynak et al. (2023) examined 331 patients who underwent total thyroidectomy for changes in TgAb pre‐surgery versus benign tumors. Despite the average age being roughly 10 years higher, confirmation was found for TgAb's prognostic impact on recurrence, matching the present study. However, contrary to our research, most patients were intermediate risk. TgAb level did not relate to risk stratification, consistent with our findings [19].

In a study by Xu et al. (2022) in which they compared 839 PTC patients with Hashimoto's thyroiditis from 2007 to 2016 in China, in terms of TgAb changes, achieving a significant postoperative decrease (≥ 50%) in TgAb 12 months following surgery portended a more favorable prognosis versus others. The results aligned with our study, differing in that we validated this antibody's value in prognosis at 6 months post‐surgery [20].

Görges et al. (2005) in Germany measured serum TgAb and Tg levels in 112 follicular thyroid cancer patients who underwent thyroidectomy and received two 131‐iodine treatments over 3 years, assessing recurrence risk. The median TgAb half‐life was 10 weeks. Contrary to our findings, this study did not support predicting TgAb course by residual tumor volume or Tg, and TgAb negativity could underestimate disease. Differences may relate to tumor type or to TgAb measurement method/duration [21].

The research by Turanli et al. (2020) in Turkey evaluated 109 PTC patients who underwent total thyroidectomy followed by lateral neck dissection and ablation between January 1989 and December 2014, and yielded contradictory results. Patients were categorized as positive or negative based on serum TgAb, comparing recurrence/persistence rates between groups. Of 101 months' follow‐up, 32 (29.3%) had recurrence, with 27/95 (28.4%) versus 5/14 (35.7%) between TgAb subgroups, thus establishing TgAb status as a useful prognostic indicator, contrasting with our findings. Discrepancies likely stem from TgAb level classification, longer follow‐up, and neglecting changes over time as in our study [22].

## 6 Limitations and Strengths

A key strength was measuring baseline TgAb levels before thyroidectomy in all patients, as pre‐surgery TgAb concentration is a more reliable control value than TgAb at ablation time, and its level is probably unstable during 131I treatment.

One potential limitation in using this study to assess recurrence risk was the lack of facilities for BRAFV600E measurement. Another limitation was some patients' unwillingness or lack of cooperation to provide additional information by completing checklists. To address this issue, researchers provided rationale for their importance. Another potential limitation is that only one TgAb assay method was utilized over the entire study period.

Although measuring Tg using the RIA method is more accurate, there is still a possibility of false‐positive TgAb results in patients with negative Tg. Further evaluations, including ultrasound, second surgery, and histopathological examination, can definitively confirm or rule out recurrence [23].

## 7 Conclusion

In this study, most of the subjects were of low‐risk type, and 6 months after surgery is a more valuable time in which to check for recurrence. In addition, tumor recurrence was best indicated by Tg and TgAb serum levels at 6 months after surgery.

## Author Contributions

**Amirian Fatemeh:** study design, data collection, literature search. **Yaghoubi Mohammad Ali:** study design, data collection. **Mehrad‐Majd Hassan:** data analysis, data interpretation. **Mohebbi Masoud:** study design, data collection. **Layegh Parvin:** study design, data collection. **Vazifeh‐Mostaan Leila:** study design, data collection. **Dadgar Moghaddam Maliheh:** data analysis, data interpretation. **Amirian Zahra:** literature search, writing. **Taghavi Ghazaleh:** literature search, writing.

## Conflicts of Interest

The authors declare no conflicts of interest.

## Data Availability

The data that support the findings of this study are available on request from the corresponding author. The data are not publicly available due to privacy or ethical restrictions.
